# Identifying and validating hypoxia- and metabolism-related hub genes and cell communication in atherosclerosis

**DOI:** 10.3389/fcvm.2025.1680482

**Published:** 2025-12-12

**Authors:** Li Hao, Qun Xu, Guang Yang, Mingxue Di

**Affiliations:** Department of Gerontology, The First Affiliated Hospital of Shandong First Medical University & Shandong Provincial Qianfoshan Hospital, Jinan, Shandong, China

**Keywords:** atherosclerosis, hypoxia, metabolism, single-cell analysis, hub genes

## Abstract

**Background:**

Atherosclerosis (AS) is a multifactorial disorder characterized by plaque formation, with hypoxia and metabolic pathways playing central roles in its pathogenesis. However, the differentially expressed metabolism-hypoxia-related genes (DE-MH-RGs) contributing to AS remain largely undefined. This study aimed to characterize DE-MH-RGs to uncover their potential regulatory mechanisms in AS.

**Results:**

Single-cell sequencing data, AS-related datasets, 200 hypoxia-related genes (HRGs), and 2,752 metabolism-related genes (MRGs) were employed for analysis. Single-cell sequencing identified distinct cell subpopulations, and differential gene expression was assessed between the AS and normal groups in GSE100927. DE-MH-RGs were determined by intersecting differentially expressed HRGs and MRGs. Functional enrichment of DE-MH-RGs was conducted through Gene Ontology (GO) and Kyoto Encyclopedia of Genes and Genomes (KEGG) analyses, followed by protein–protein interaction (PPI) network construction. Hub genes were identified using least absolute shrinkage and selection operator (LASSO) logistic regression and further validated by gene set enrichment analysis (GSEA) and Gene Set Variation Analysis (GSVA). Immune feature analysis assessed immune cell proportions and identified potential drug candidates. Hub genes were also pinpointed in single-cell data (GSE159677) using ReactomeGSA, cell communication, and pseudotime analyses. Single-cell analysis revealed five distinct subpopulations—T lymphocytes, endothelial cells, macrophages, vascular smooth muscle cells (VSMCs), and B lymphocytes—and 141 DE-MH-RGs. Enrichment analysis highlighted the association of these genes with circulatory system pathways, including vascular functions and responses to oxygen and hypoxia. The hub genes, RYR2, ABCC9, KCNJ2, EGFR, and SLC7A8, exhibited strong diagnostic potential. GSVA analysis linked the hub genes to several metabolic pathways. Thirteen immune cell types showed significant differences between normal and AS samples. Potential therapeutic agents, such as afatinib, were identified. The hub genes were primarily located in VSMCs. Most AS cells displayed low differentiation levels.

**Conclusions:**

Transcriptomic data from AS identified key hub genes related to hypoxia and metabolism, offering valuable insights for future research.

## 1 Background

Atherosclerosis (AS) is a chronic vascular condition characterized by the accumulation of lipid-rich plaques in the arterial intima, which can lead to cardiac ischemia and cerebrovascular events, significantly impacting global morbidity and mortality rates (1, 2). A key phase in AS progression involves sustained inflammation, marked by the deposition of low-density lipoprotein (LDL) particles in major arteries, migration of monocytes and immune cells through damaged endothelial layers, smooth muscle cell proliferation, and plaque formation (3). Beyond statins, emerging therapies such as inclisiran (PCSK9 monoclonal antibody) and canakinumab [interleukin-1β (IL-1β) monoclonal antibody] have been successfully administered to high-risk patients with AS, yielding promising results (1, 4). However, given the limitations of current AS treatments (5), it is essential to explore the molecular mechanisms regulating cell functions in vulnerable plaques, identify potential therapeutic targets, inhibit plaque inflammation, and enhance plaque stability to advance prevention, diagnosis, and treatment strategies for AS.

Hypoxia, a prevalent stressor, exerts significant effects on aerobic organisms, influencing conditions such as bacterial infections, inflammation, cardiovascular diseases, and cancer (6, 7). In response to low oxygen levels, human physiology adapts by regulating cellular protein synthesis, energy metabolism, mitochondrial function, lipid and carbohydrate processing, and nutrient absorption, all of which are crucial for effectively managing hypoxic stress (8, 9). Hypoxia frequently occurs within atherosclerotic plaques during disease progression, significantly impacting plaque development and vulnerability (10). Several factors contribute to hypoxia-related AS. Within atherosclerotic plaques, hypoxia activates hypoxia-inducible factor 1α (HIF-1α), driving the expression of genes linked to angiogenesis, metabolism, and inflammation (11). Additionally, hypoxia-mediated upregulation of inflammatory signaling pathways recruits and activates immune cells, such as monocytes/macrophages, within the plaque microenvironment, exacerbating inflammation and promoting plaque growth and instability (12). Furthermore, angiogenesis worsens plaque progression (13). Hypoxia enhances LDL uptake by macrophages and disrupts cholesterol efflux, leading to lipid accumulation and foam cell formation (14). Under hypoxic conditions, vascular smooth muscle cells (VSMCs) shift from a contractile to a synthetic phenotype, increasing proliferation and extracellular matrix production, which facilitates plaque expansion and fibrous cap formation. However, this shift can also induce VSMC apoptosis, weakening the fibrous cap and increasing the risk of plaque rupture (15).

Obstructive sleep apnea (OSA), a common condition, may also contribute to AS pathogenesis. The reoxygenation following hypoxia triggers oxidative stress via the generation of reactive oxygen species (ROS), which in turn induces inflammation, endothelial dysfunction, and metabolic abnormalities (16). Obesity, often associated with metabolic syndrome, promotes the production of cytokines such as tumor necrosis factor-alpha (TNF-α), IL-1β, and IL-6, further contributing to AS. Metabolic factors such as lipids, uric acid, bile acids, and cholesterol metabolism play critical roles in the initiation and progression of AS (17, 18). Despite these insights, the specific roles of hypoxia metabolism-related genes (HM-RGs) in AS remain unclear and warrant further investigation.

Utilizing multiple single-cell and transcriptome datasets related to hypoxia and metabolism, along with machine learning techniques and other analytical approaches, this study aims to elucidate the relationships between hypoxia, metabolism, and AS. Hub genes associated with hypoxia metabolism in AS were identified, their related biological pathways analyzed, and their potential regulatory mechanisms, along with associated drugs, explored. Additionally, the distribution and expression of these hub genes in single-cell datasets were examined to enhance our understanding of the underlying mechanisms of AS, hypoxia, and metabolism, thereby laying the groundwork for the development of novel targeted therapies.

## 2 Methods

### 2.1 Data collection

**Figure 1:**
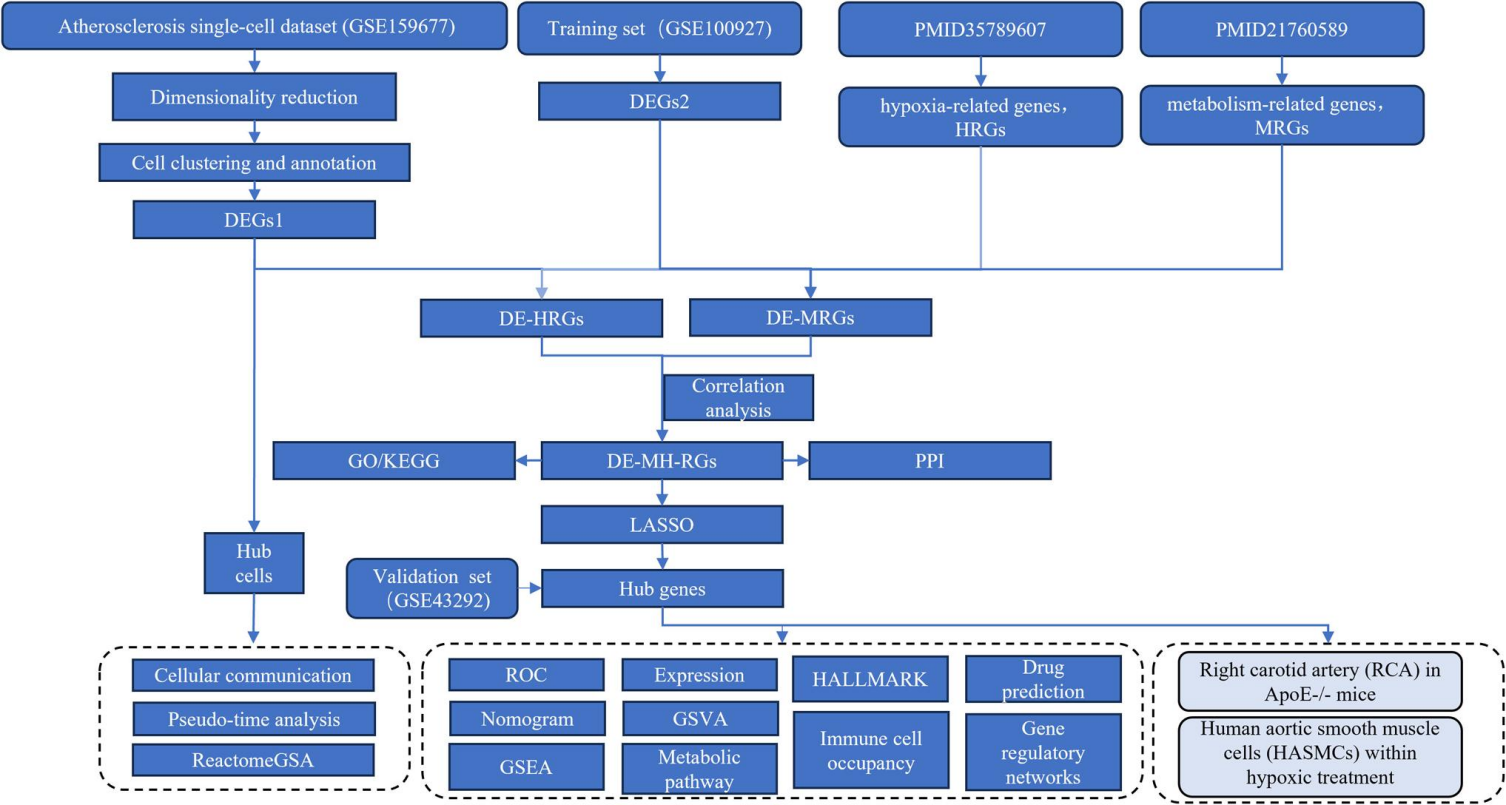
Flowchart showing the progression of the study. The single-cell dataset (GSE159677) and AS-related datasets (GSE100927 and GSE43292) were sourced from the GEO database for analysis.

The single-cell dataset (GSE159677) and AS-related datasets (GSE100927 and GSE43292) were obtained from the GEO database (https://www.ncbi.nlm.nih.gov/geo/). GSE159677 includes 3 carotid artery tissue samples from patients with AS and 3 matched proximal adjacent tissue samples from the same patients. In GSE100927, AS carotid artery tissue samples and 12 normal carotid artery samples were used as the training set. The testing set, GSE43292, comprised 32 AS carotid artery tissue samples and 32 normal carotid artery samples. The research team extracted 200 hypoxia-related genes (HRGs) from the Molecular signatures database (MSigDB) (https://www.gsea-msigdb.org/gsea/msigdb), primarily selected from the HALLMARK_HYPOXIA gene set (ID: M5891), and obtained 2,752 metabolism-related genes (MRGs) from previously published studies (19). Bioinformatics approaches were employed to analyze the biological features of AS, as outlined in Figure 1.

### 2.2 Normalization of single-cell sequencing data followed by PCA downscaling

Single-cell sequencing data were processed using the CreateSeuratObject function from Seurat (v4.3.0) (20) with parameters (min.cells = 3, min.features = 200). Mitochondrial gene expression was quantified using the percentage feature set function, and cells with more than 10% mitochondrial gene content were excluded. Data features were visualized using violin plots. Data normalization was performed using the NormalizeData function, while highly variable genes were identified using the FindVariableFeatures function, facilitating cell type identification. Principal component analysis (PCA) was employed to reduce data dimensionality, selecting 30 principal components prior to analysis based on the inflection points.

### 2.3 Cell clustering and annotation

Unsupervised clustering of cells was performed using the FindNeighbors and FindClusters functions from the Seurat package. Marker genes for each cell group were identified using the FindAllMarkers function (min.pct = 0.6, only.pos = TRUE, logfc.thresh = 0.5) with the Wilcoxon method and a threshold of 0.5. The marker genes were compared with those in the CellMarker database to identify cell subpopulations, and the singleR algorithm and references were used to assist in cell type identification. Specifically, annotations were made according to reference (21) (Table 1). The distribution of these cell subpopulations was visualized via t-SNE clustering. Dot plots and bar charts illustrating the markers and proportions of each subpopulation were created using the “ggplot2” package (v3.4.1) (22).

**Table 1:** List of cell makers.

Cell type	Marker gene
T−Lymphocytes	CD2, TRAC, CD69
Macrophage	AIF1, CD14, CD68
VSMCs	CALD1, MYL9, TAGLN
Endothelial	VWF, PECAM1, ECSCR
B−Lymphocytes	CD79A, MS4A1, IGKC

### 2.4 Differential expression analysis

To identify disease state-related differentially expressed genes, we performed differential analysis at the single-cell level. First, for the GSE159677 single-cell dataset, we used a pre-normalized gene expression matrix. The normalization had previously been completed using the NormalizeData function from the Seurat package with the “LogNormalize” method, which involved normalizing the gene expression counts per cell to 10,000, followed by natural logarithm transformation [formula: log1p(UMI counts/total UMI counts × 10,000)], thereby correcting for differences in sequencing depth between cells. Based on this normalized data, we used the FindMarkers function in the R package “Seurat” to perform differential gene expression analysis between different samples (KD vs. Control) across the annotated cell types (threshold: |average log2FC| > 0.5, adj.*p*.value < 0.05). We generated Manhattan plots to display the differential genes between samples across different cell types, and the union of these differentially expressed genes from different cell types between samples was defined as significant cell-related genes. For comparison, we also analyzed the bulk transcriptomic dataset GSE100927. Differential genes between AS patients and normal controls were identified using the “limma” package (v3.46.0) (23), with the criteria of adj.*p*.value < 0.05 and |log2FoldChange| > 0.5. The results were visualized using the “ggplot2” package (v3.4) (22) and the “ComplexHeatmap” package (v2.12.1) (24). To subsequently focus on metabolism-hypoxia cross-talk genes, we designated the DEGs from the single-cell dataset as DEGs1 and those from the bulk dataset GSE100927 as DEGs2. By intersecting DEGs1 and DEGs2 with metabolism-related genes and hypoxia-related genes, respectively, we obtained differentially expressed metabolism-related genes and differentially expressed hypoxia-related genes. These intersection results were visualized using Venn diagrams generated with the “ggVennDiagram” package (v1.2.2) (https://CRAN.R-project.org/package=ggVennDiagram). Subsequently, we performed pairwise Spearman correlation analysis on the two sets of differential genes, applying a filtering threshold of |cor| > 0.8 and *p* < 0.05. Genes meeting these criteria were defined as the final differentially expressed metabolism-hypoxia related genes (DE-MH-RGs) (25).

### 2.5 Analysis of functional enrichment and protein–protein interactions (PPIs)

Gene Ontology (GO) and Kyoto Encyclopedia of Genes and Genomes (KEGG) analyses were performed using the “clusterProfiler” (v4.4) (26) and “org.Hs.eg.db” (v3.15.0) (27) packages. Functional annotation of DE-MH-RGs was conducted with a significance threshold of *p* adj < 0.05. The results for each major category were visualized using dendrograms generated by the “ccgraph” (v1.0.2) (28) package. To explore the relationships among DE-MH-RGs, a PPI network was constructed through the STRING database (https://string-db.org), excluding proteins with a confidence score below 0.4. These findings were visualized using Cytoscape (v3.7.1) (29). The PPI network was modularized using the “MCODE” plugin to identify key modular genes, applying the following settings: Loops: false, Degree Cutoff: 2, Node Score Cutoff: 0.2, Haircut: true, Fluff: false, K-Core: 2, and Max. Depth from Seed: 100.

### 2.6 Determination of hub genes

Hub genes were identified using least absolute shrinkage and selection operator (LASSO) logistic regression analysis of key modular genes from the GSE100927 dataset. Receiver operating characteristic (ROC) curves were constructed with pROC (v1.17.0.1) (30) to assess the diagnostic significance of the hub genes in the GSE100927 and GSE43292 datasets. The expression profiles of the hub genes were analyzed with the “ggpubr” package (v0.4.0) (31).

### 2.7 Clinical application and pathway analysis of the hub genes

A nomogram was developed using the “rms” package (v6.2-0) (32) to evaluate the clinical AS prevalence risk based on hub gene analysis. ROC curves were utilized to assess the predictive performance of the nomogram, and decision curve analysis (DCA) was conducted using the “rmda” package (v1.6) (33). Gene set enrichment analysis (GSEA) was performed with the “clusterProfiler” (v4.4.4) (26) and KEGG database to explore pathways associated with key genes. The correlation coefficients of hub genes with other genes were calculated, ranked, used for GSEA, and visualized with the GSEA ridge map using “GseaVis” (v0.0.5) (34).

### 2.8 Gene set variation analysis (GSVA)

In GSE100927, KEGG pathway scores for each sample were calculated using GSVA (v1.38.2) (35). A comparative analysis of KEGG pathways was conducted between the AS and NC groups using the “limma” package (v3.46.0) (23) (*p* adj < 0.05). The top 20 pathways that were significantly upregulated or downregulated were visualized in heatmaps generated using the “pheatmap” package (v1.0.12) (24) and bar charts created with “ggplot2” (v3.4.21) (22). Spearman correlation analysis was performed to evaluate hub gene correlations, and the “ggplot2” package was used to visualize the top 10 metabolism-related pathways with significant regulation (|cor| > 0.3, *p* < 0.05).

HALLMARK inflammatory pathway scores were calculated using GSVA. Key genes were analyzed for Spearman correlation with these pathways and visualized through scatterplots generated with the “ggExtra” package (v0.10.0) (https://CRAN.R-project.org/package=ggExtra) (|cor| > 0.3, *p* < 0.05). Circle plots illustrating the correlation analysis of hub genes were generated using the “circlize” package (v0.4.15) (24). The functional similarity of the hub genes was assessed using “GOSemSim” (v2.24.0) (36).

### 2.9 Immune cell occupancy and gene regulatory networks

The proportions of immune cell types in all samples from GSE100927 were evaluated using the CIBERSORT algorithm, with a significance threshold of *p* < 0.05. Immune cell correlations were assessed via a heatmap generated with the “corrplot” package (v0.92). The rank sum test was applied to compare immune cell proportions between the NC and AS groups, with variations in immune cell types visualized using box plots via the “ggpubr” package (v0.4.0) (29) (*p* < 0.05). Spearman correlation analysis was performed to examine the relationships between hub genes and distinct immune cells (|cor| > 0.3, *p* < 0.05).

A regulatory network involving lncRNAs, miRNAs, and mRNAs was constructed using the miRWalk database (http://mirwalk.umm.uni-heidelberg.de/) to predict miRNAs targeting hub genes. The starBase database (https://ngdc.cncb.ac.cn/) was used to identify relationships between lncRNAs and miRNAs. Hub gene-targeting transcription factors (TFs) were predicted via the NetworkAnalyst database (https://www.networkanalyst.ca/).

### 2.10 Prediction of drugs and hub gene expression levels in each cell subpopulation

The DGIdb database (http://dgidb.genome.wustl.edu/) was employed to predict drugs that target the hub genes. t-SNE algorithms were used to visualize hub gene expression across cell subpopulations, with a dot plot displaying hub gene expression within each group. Cells with significant differential expression of all hub genes were identified as key cells.

### 2.11 Cellular communication and differentiation

Individual cell function enrichment analysis was performed using the “ReactomeGSA” package (v1.4.2) (37), with the top 15 pathways displayed in a heatmap. The KEGG metabolic pathway score and subpopulation comparisons were analyzed using the “GSVA” package (v1.38.1) (35). Cellular communication analysis was conducted with CellPhoneDB, a leading software for intercellular ligand-receptor interactions (*p* < 0.05, means > 2). The differentialGeneTest function was used to extract key cell data and identify DEGs between the AS and NC groups. Pseudotime analysis was performed using Monocle (v2.18.0) (37).

### 2.12 Pseudotime analysis

To clarify the differentiation trajectory of key cells during disease progression, the key cells were first subjected to secondary clustering. The R package “Seurat” was used to normalize the key cells using the ScaleData function. Subsequently, principal component analysis (PCA) was performed on highly variable genes using the RunPCA function for dimensionality reduction. The elbow plot and JackStraw plot were combined to determine the principal components for subsequent analysis. The FindClusters function in the Seurat package (resolution set to 0.05) was then applied to obtain distinct cell clusters, and the t-SNE plot was used for cluster visualization.

Next, the FindAllMarkers function in the Seurat package (parameters: min.pct = 0.25, logfc.threshold = 0.05, test.use = “wilcox”, *p* adj < 0.05) was employed to identify specific marker genes for each cell cluster. The ClusterGVis package (v:0.1.4) (38) was used to perform KEGG pathway enrichment analysis on these marker genes (the top five most significantly enriched pathways were displayed for each cell type). Cell clusters containing early activated pathways were identified as the starting point of differentiation (39). Finally, pseudotime analysis was conducted using the R package Monocle (v2.18.0) (37). During the analysis, the pseudotime trajectory and systematic expression changes of key genes were simultaneously examined to validate the biological significance of the differentiation trajectory.

### 2.13 Atherosclerosis animal model protocol

For *in vivo* validation, eight-week-old male ApoE^–/–^ mice were fed a high-fat diet containing 0.25% cholesterol and 15% cocoa butter (Beijing HFK Bioscience Co., Ltd.) for 12 weeks to establish an AS model. Silica collars were applied to the right carotid artery (RCA) during the third week to promote the development of atherosclerotic lesions. Anesthesia for collar placement was induced with an intraperitoneal injection of 40 mg/kg sodium pentobarbital. After 12 weeks, the mice were euthanized with an intraperitoneal injection of 100 mg/kg sodium pentobarbital, perfused with PBS via the abdominal aorta, and then exsanguinated.

### 2.14 Immunohistochemistry

The RCAs and LCAs were dissected, fixed, embedded, and sectioned into 5 μm-thick slices. Cryosections were incubated with 5% BSA, followed by overnight incubation with primary antibodies at 4°C. Detection was performed using an HRP system (ZSGB-BIO) and 3,3′-diaminobenzidine (DAB) (ZSGB-BIO). Plaque staining was visualized using a color CCD video microscope (Olympus, Japan) and analyzed with Image-Pro Plus 6.0 software (Media Cybernetics, USA).

### 2.15 Hypoxic treatment

Human aortic smooth muscle cells (HASMCs) (ScienCell Research Laboratories, Carlsbad, CA, USA) were cultured in smooth muscle cell-specific medium from the same supplier. Cells were cultured to 80% confluency at 37°C with 5% CO2% and 21% O2, and then continued to be cultured for 48 h under normoxia(21% O2), mild hypoxia(10% O2) or severe hypoxia (1% O2).Then cells were washed with PBS, and collected for qRT–PCR. Experiments were performed with primary cells at passages 4–8.

### 2.16 RNA isolation and qRT–PCR

Total RNA was extracted from HASMCs using TRIzol reagent (Ambion, Life Technologies, USA) and reverse-transcribed into cDNA with a cDNA Synthesis Kit (R211-01, Vazyme, China). For qPCR, 1 ng of cDNA was amplified using SYBR Green (Q511-02, Vazyme, China) to quantify mRNA expression. The 2^–*ΔΔ*Ct^ method was employed for quantification, with normalization to *β*-actin mRNA levels.

### 2.17 Statistical analysis

Bioinformatic analyses were performed using R software (v 4.2.2). Statistical analysis was performed with SPSS v24.0 (SPSS Inc., Chicago, IL) and Prism 8.0 (GraphPad, USA), presenting results as means ± SEM from at least three independent experiments. Comparisons were made using Student's t-test, with a significance threshold set at *p* < 0.05.

## 3 Results

### 3.1 T lymphocytes, endothelial cells, macrophages, VSMCs, and B lymphocytes were identified

**Figure 2:**
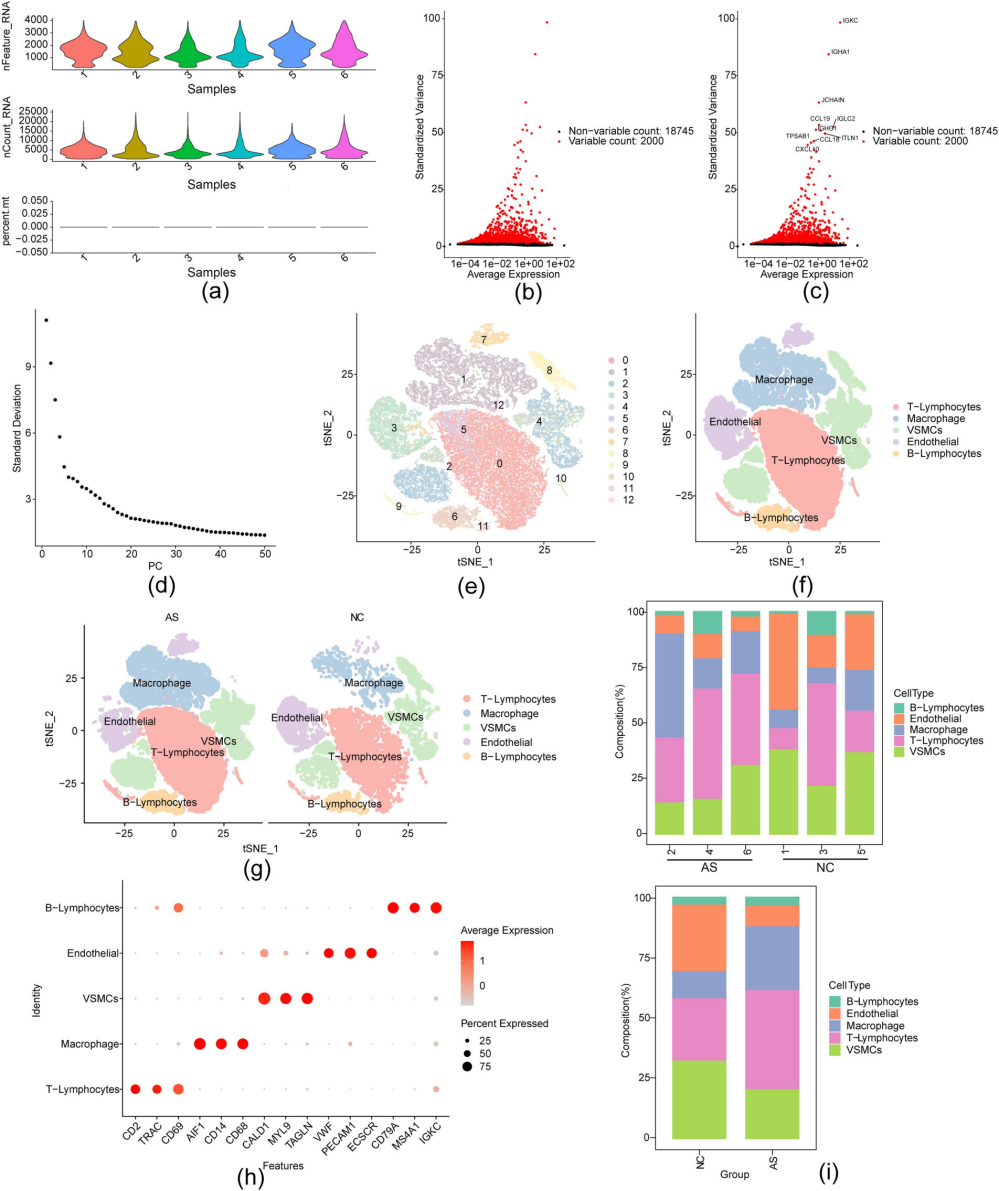
The scRNA-Seq identified T-lymphocytes, endothelial, macrophage, VSMCs, B-lymphocyte identification of five distinct cell populations in atherosclerotic carotid. **(a)** Violin plot of single-cell sequencing data features; **(b,c)** Intercellular expression of highly variable genes; **(d)** Principal component analysis (PCA) reduces data dimensionality and generates an inflection point plot for the selected data's available dimensions; **(e)** unsupervised cluster analysis; **(f,g)** Tsne cell cluster distribution map of cell subsets visualization; **(h)** Expression plot of marker genes across the five clusters; **(i)** Bar chart of cell proportion between different samples and different groups.

Preprocessing of the GSE159677 single-cell sequencing dataset identified 20,745 genes and 50,370 cells. The AS group contained 38,329 cells, whereas the NC group contained 12,041 cells (Figure 2a). After normalization, 2,000 highly variable genes were selected using default parameters (Figures 2b,c). Dimensionality reduction via PCA was performed, and the top 30 principal components were retained for subsequent clustering analysis (Figure 2d). The results of the unsupervised clustering are shown in Figure 2e. Following manual annotation, 13 clusters were identified, representing five cell subpopulations: T lymphocytes, endothelial cells (ECs), macrophages, VSMCs, and B lymphocytes (Figures 2f,g). Dot plots revealed T lymphocyte-specific and AIF1 markers for macrophages, while bar charts showed a lower proportion of B lymphocytes and ECs in each GSE159677 sample (Figures 2h,i).

### 3.2 A total of 141 DE-MH-RGs were identified

**Figure 3:**
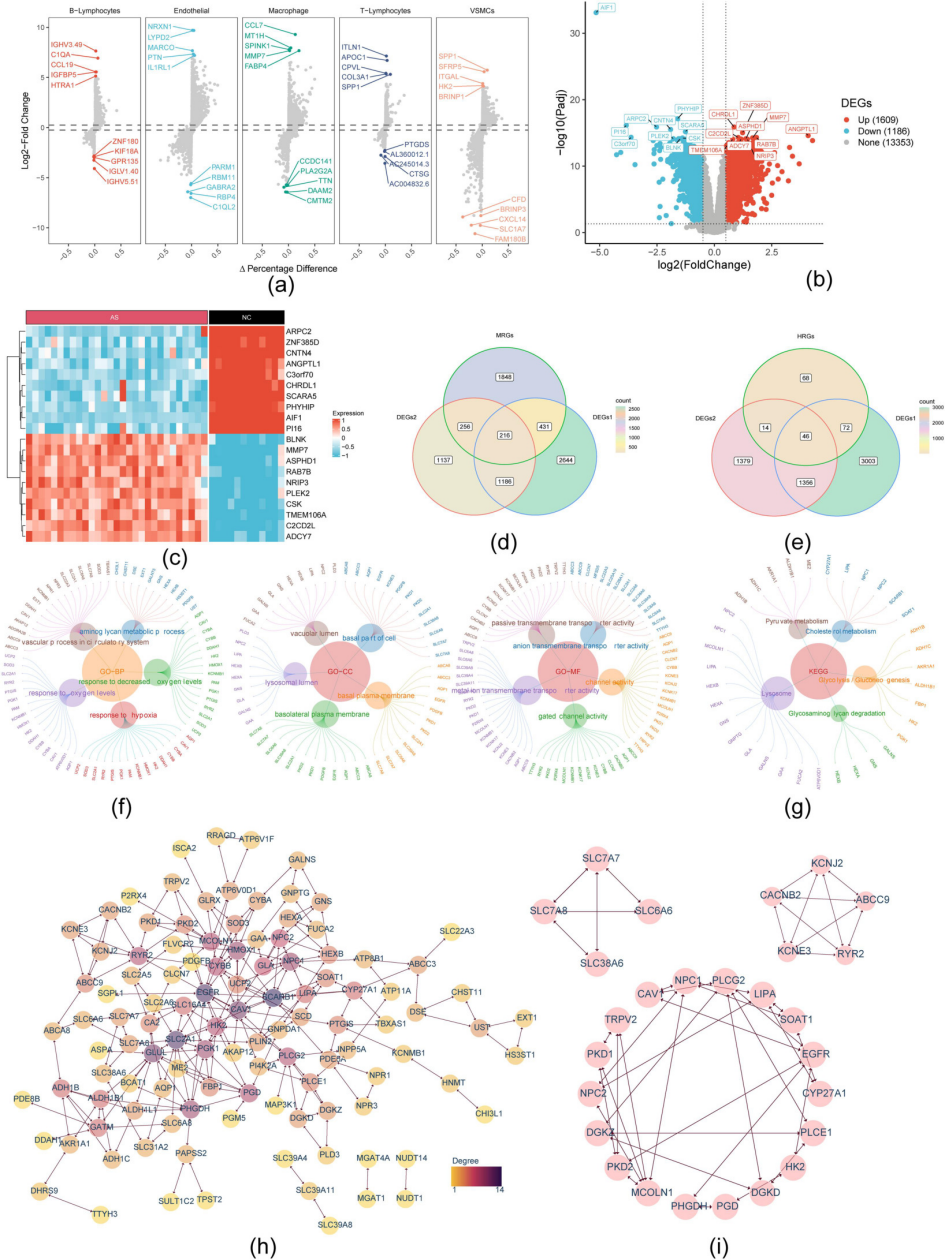
Transcriptional profiles and functional enrichment analysis of distinct cells in atherosclerotic carotid. **(a)** Single cell data differential gene volcano map (DEG1); **(b)** Volcano plot of differentially expressed genes between AS and NC in the GSE100927 database, with the *x*-axis representing the log2 Fold Change and the *y*-axis representing the -log10 (padj) significance.(padj < 0.05, |log2FoldChange| > 0.5), The TOP10 genes up/down-regulated were labeled (DEG2); **(c)** Heat map of the top 10 up-regulated genes differentially expressed between AS and NC; **(d)** Venn diagram illustrating the overlap between differentially expressed genes (DEG1 and DEG2) and metabolism-related genes (MRGs); **(e)** Venn-diagram on overlap of differentially expressed genes of DEG1, DEG2 and hypoxia-related genes (HRGs); **(f)** The Gene Ontology (GO) analysis bar plot illustrated the enrichment of DE-MH-RGs in biological processes (BP), cellular components (CC), and molecular functions (MF); **(g)** The KEGG-gene analysis plot illustrates the DE-MH-RGs; **(h)** Protein interaction network of differential DE-MH-RGs genes; **(i)** The PPI network was segmented into three modules using MCODE, yielding 27 key module genes for further analysis.

In the GSE159677 dataset, we identified DEGs across five cell types. The results showed that 2,541 differential genes were identified in Endothelial cells, 1,841 in VSMCs, 1,179 in Macrophages, 340 in T-Lymphocytes, and 113 in B-Lymphocytes. After deduplication, these cell type-specific differential genes yielded a total of 4,477 unique genes, comprising 3,066 upregulated and 1,850 downregulated genes (Figure 3a). The GSE100927 dataset revealed 2,216 DEGs, with 977 upregulated and 1,239 downregulated genes (Figures 3b,c). After intersecting DEGs1 and DEGs2 with MRGs and HRGs, 216 DE-MRGs and 46 DE-HRGs were identified (Figures 3d,e). Combining these DEGs, 141 DE-MH-RGs were identified (Supplementary Table S1), primarily associated with vascular processes in the circulatory system, lysosomal lumen, and metal ion transmembrane transporter activity (Figure 3f). KEGG functional enrichment analysis showed that DE-MH-RGs were linked to lysosomal, glycolysis/glyconeogenesis, and other pathways (Figure 3g). These DE-MH-RGs were significantly enriched in circulatory system functions and glucose metabolism. PPI analysis of the 141 DE-MH-RGs revealed 112 protein-interaction relationships (Figure 3h). Module analysis using the mode plugin identified 27 key genes in three modules (Figure 3i).

### 3.3 KCNJ2, SLC7A8, EGFR, ABCC9, and RYR2 were considered hub genes

**Figure 4:**
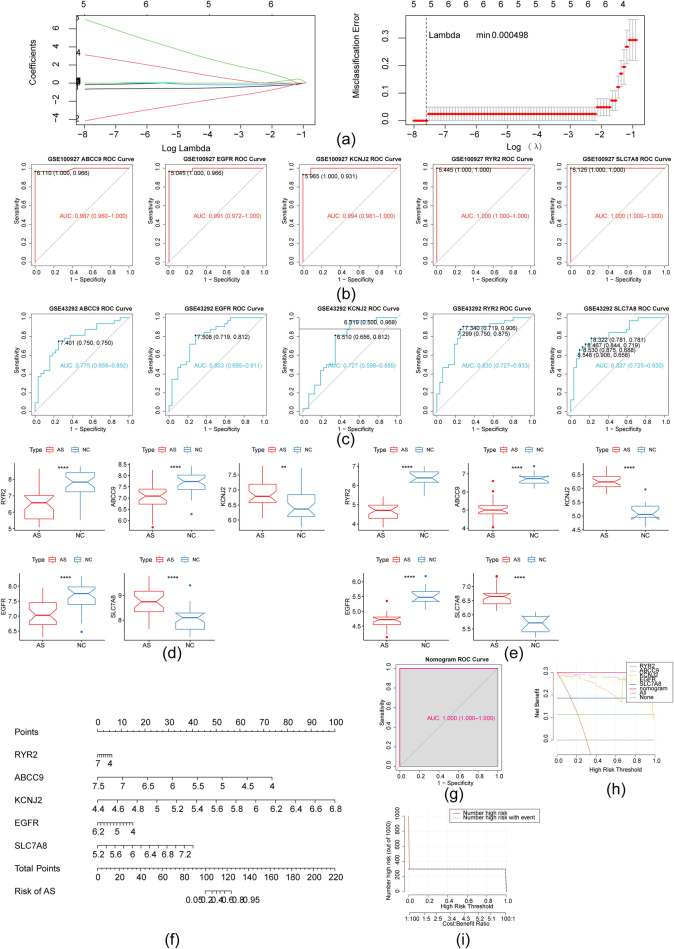
Development and validation of a diagnostic model using five hub genes. **(a)** Using the LASSO method, 5 DE-MH-RGs were identified as diagnostic biomarkers; **(b)** ROC curves assessed the diagnostic efficacy of five genes in GSE100927, yielding AUC values of 0.987 for ABCC9, 0.991 for EGFR, 0.994 for KCNJ2, and 1.000 for both RYR2 and SLC7A8; **(c)** ROC curve analysis of the test set (GSE43292) for the diagnostic model incorporating the five genes yielded the following AUC values: ABCC9 (0.775), EGFR (0.803), KCNJ2 (0.727), RYR2 (0.830), and SLC7A8 (0.827); **(d)** Box plot of the expression levels of five hub genes in dataset GSE100927; **(e)** Box plot illustrating the expression of five hub genes in dataset GSE43292.(*p < 0.05, **p < 0.01, ***p < 0.001, ****p < 0.0001); **(f)** A nomogram was developed to estimate the incidence of carotid atherosclerosis. The total score, derived from individual index values, provided the predicted AS risk rate according to the Nomogram; **(g)** Training set Nomograms -ROC curve; **(h)** DCA curve for evaluating the clinical utility of the nomogram model; **(i)** Clinical impact curve of nomogram model.

LASSO regression analysis identified five hub genes: RYR2, ABCC9, KCNJ2, EGFR, and SLC7A8 (Figure 4a). ROC curve analysis in the GSE100927 dataset demonstrated strong diagnostic potential for all five hub genes, which was confirmed in the GSE43292 dataset (Figures 4b,c). In both GSE100927 and GSE43292 datasets, expression of EGFR, ABCC9, and RYR2 was decreased in AS samples, whereas KCNJ2 and SLC7A8 expression levels were increased (Figures 4d,e). These five hub genes (KCNJ2, SLC7A8, EGFR, ABCC9, and RYR2) were further investigated. A nomogram was constructed based on these hub genes, and the ROC curve AUC indicated strong predictive performance (Figures 4f,g). The nomogram model exhibited superior clinical advantage over the hub gene-based models in the DCA curve (Figure 4h). Additionally, the nomogram demonstrated greater accuracy in predicting outcomes, as shown in the clinical impact curve (Figure 4i).

### 3.4 Hub genes associated with metabolic pathways and inflammatory pathways

**Figure 5:**
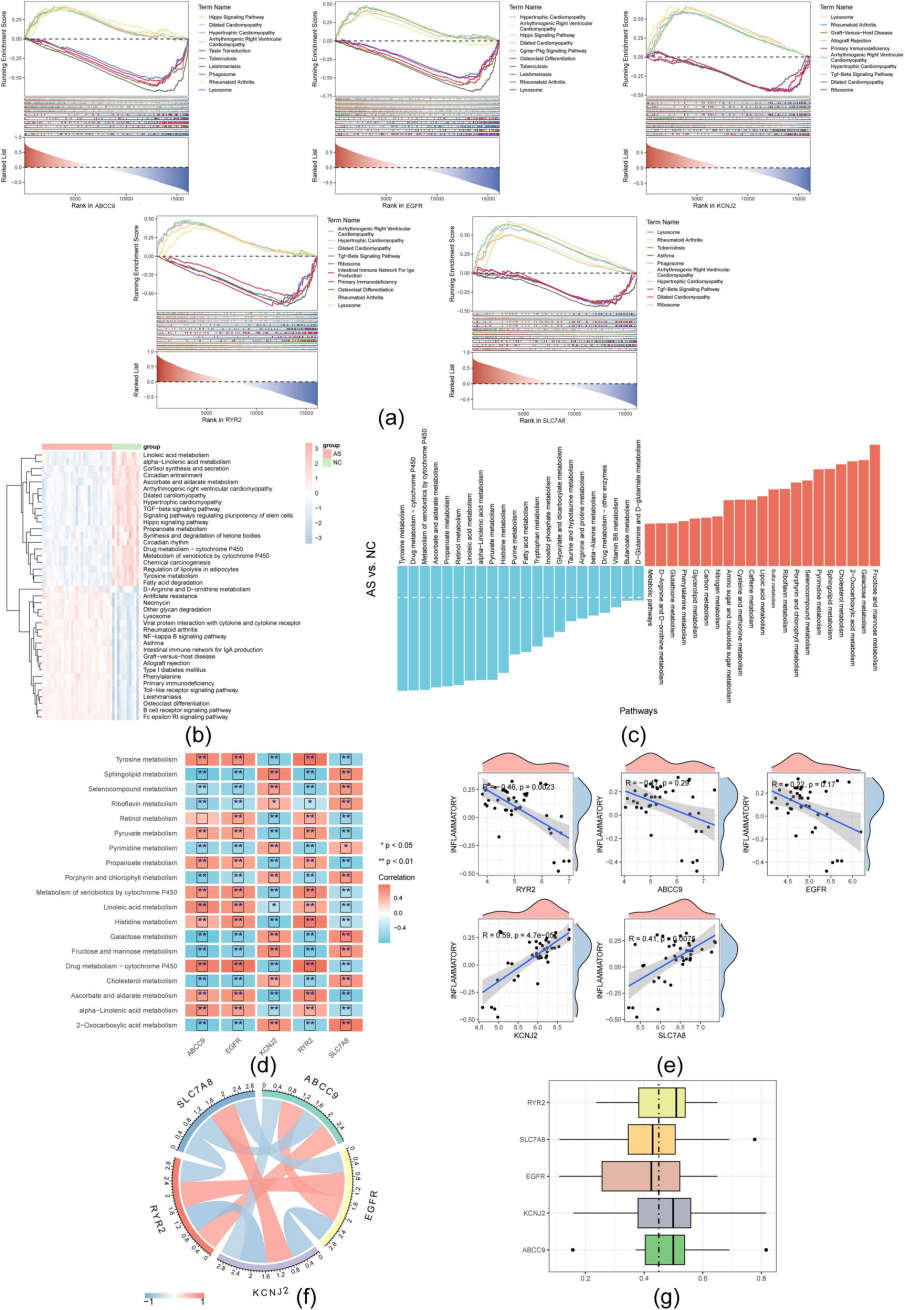
Hub genes were associated with metabolic pathways and inflammatory pathways. **(a)** GSEA ridge diagram for the five hub genes and visualization of TOP 5 pathways; **(b)** GSVA analysis of the top 20 upregulated and downregulated pathways in AS and NC groups; **(c)** Histogram of variations in metabolism-related pathways; **(d)** Heat map depicting the correlation between five hub genes and the ten most notably upregulated and downregulated genes in metabolism-related pathway; **(e)** Scatter plot illustrating the correlation between five hub genes and inflammatory pathways; **(f)** The correlation loop graph among 5 hub gene; **(g)** Functional similarity of hub genes.

GSEA ridge plots identified the top 5 pathways associated with the hub genes (Figure 5a). The heatmap revealed activation of the Hippo and TGF-β signaling pathways, along with the synthesis and degradation of ketone bodies, while propanoate metabolism, cortisol synthesis and secretion, and circadian entrainment were inhibited (Figures 5b,c). Multiple correlations were found between the hub genes and metabolism-related pathways. Specifically, RYR2, ABCC9, and EGFR showed negative correlations with inflammatory pathways, while KCNJ2 and SLC7A8 exhibited positive correlations (Figures 5d,e). Strong correlations and functional similarities were also detected among the five hub genes (Figures 5f,g).

### 3.5 Multiple correlations were detected between immune cells and hub genes

**Figure 6:**
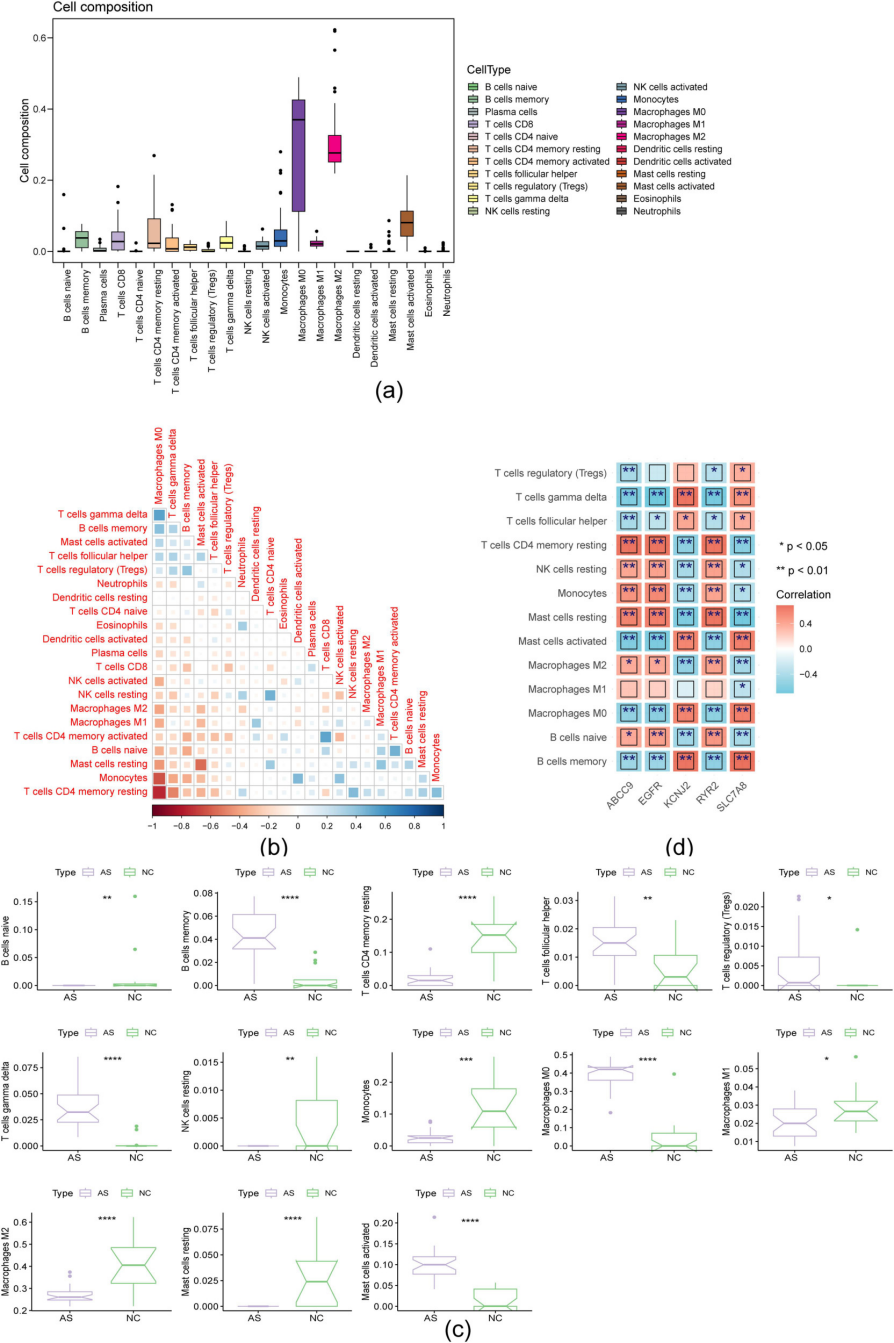
Multiple correlations were existed in immune cells and hub genes. **(a)** Box diagram of immune cell compositions; **(b)** Heat map illustrating immune cell correlation; **(c)** Box diagram illustrating the variation in proportions of immune cells. (*p < 0.05, **p < 0.01, ***p < 0.001, ****p < 0.0001); **(d)** Heat map depicting the correlation between hub genes and various immune cells.

The box plot demonstrated a relatively high proportion of macrophages across all samples (Figure 6a). Immune cell interactions were observed, with resting dendritic cells strongly associated with activated mast cells, and regulatory T cells (Tregs) significantly correlated with activated memory CD4T cells (Figure 6b). Significant differences in the proportions of 13 immune cell types were found between the NC and AS groups (Figure 6c). Compared to the NC group, monocytes made up a smaller percentage in the AS group, whereas M0 macrophages were more prevalent. Spearman correlation analysis revealed a significant positive correlation between SLC7A8 and B-cell memory, and a pronounced negative correlation between EGFR and gamma delta T cells (Figure 6d).

### 3.6 Regulatory networks and potential drugs associated with the hub genes

**Figure 7:**
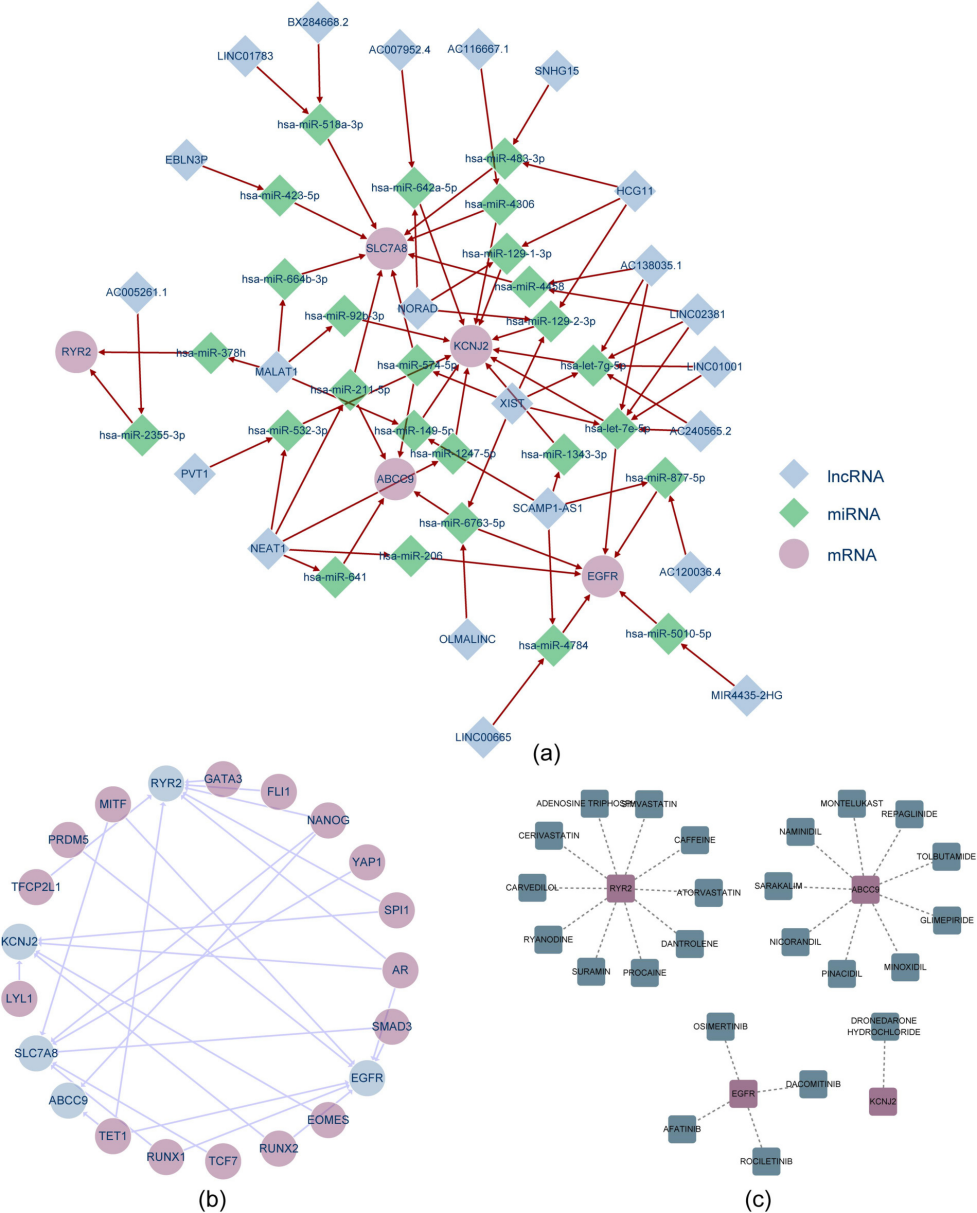
Regulatory networks and potential drugs targeting hub genes. **(a)** Regulatory circuitry of hub genes encompassing lncRNA, miRNA, and genes; **(b)** TF-gene regulatory network of hub genes; **(c)** Hub gene-drug network map via DGIdb database.

A regulatory network involving lncRNAs, miRNAs, and mRNAs was constructed to predict the miRNAs associated with the hub genes, comprising 5 hub genes, 26 miRNAs, and 22 lncRNAs, forming a total of 77 relationship pairs (Figure 7a). Specific relationship pairs, such as hSLC7A8-hsa-miR-518a-3p-LINC01783 and ABCC9-hsa-miR-6763-5p-OLMALINC, were identified. Additionally, a TF-mRNA regulatory network predicted the TFs targeting the hub genes, including 5 hub genes and 16 TFs, forming a total of 27 relationship pairs (Figure 7b). SPI1 was linked to RYR2 and KCNJ2, and TET1 was associated with ABCC9 and EGFR. Cardiovascular disease-related drugs, such as afatinib, rociletinib, and simvastatin, were identified as potential treatments for hub genes via the DGIdb database (Figure 7c).

### 3.7 Single-cell expression of hub genes and intercellular communication

**Figure 8:**
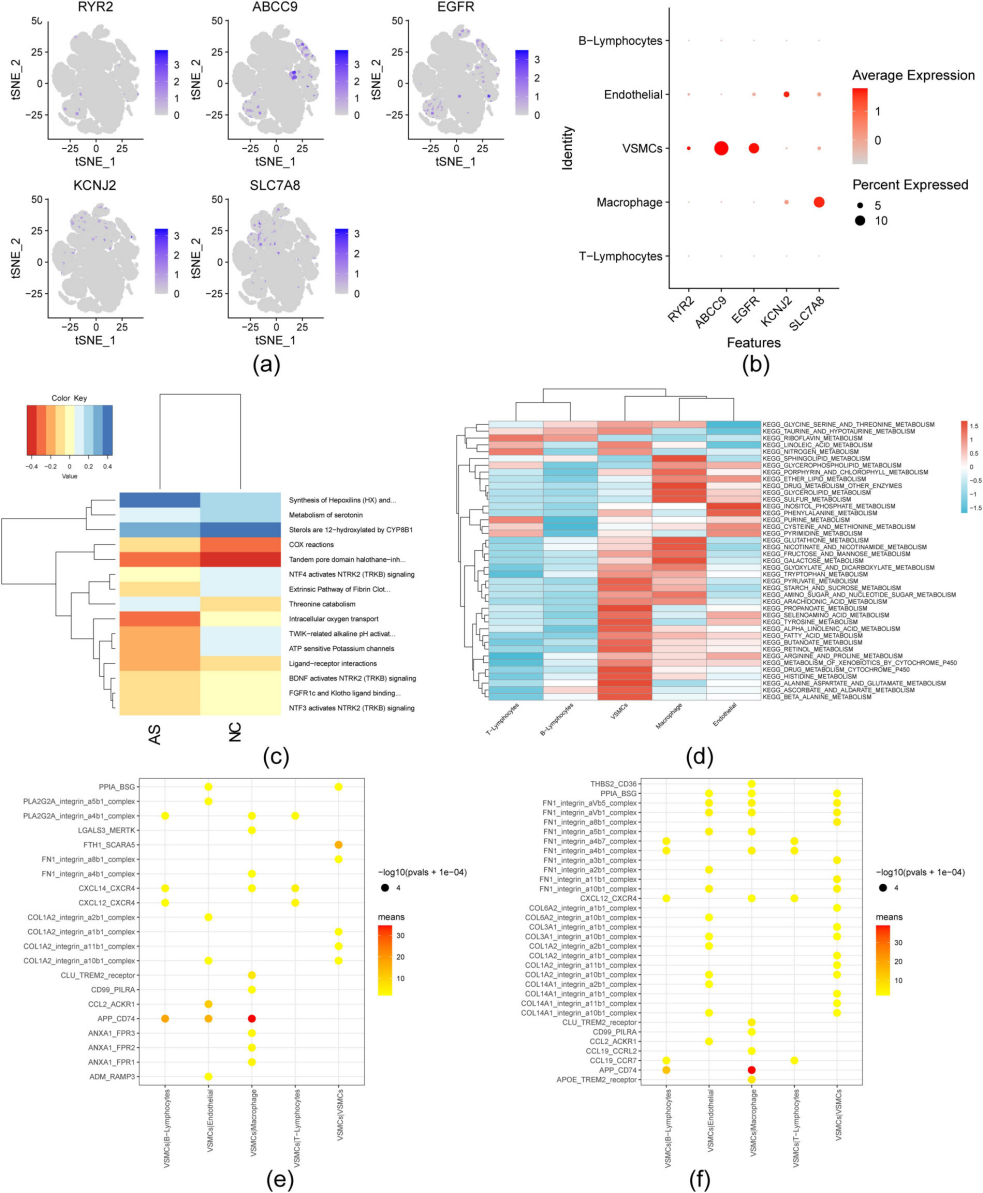
Expression of hub genes within single cells and cellular communication. **(a)** Visualization of hub genes via t-SNE; **(b)** Dot plot illustrating hub gene expression across five cell types; **(c)** Heatmap for top 15 Reactome pathways between NC and AS cells; **(d)** Heat Map for KEGG Metabolic Pathways; **(e)** VSMCs - other cell receptor ligands in NC group; **(f)** VSMCs - other cell receptor ligands in AS group.

VSMCs, as key cells in both the AS and NC groups, were analyzed due to their increased expression of hub genes (Figures 8a,b). Cellular functional enrichment analysis revealed significant enrichment of serotonin and sterol metabolism pathways, particularly 12-hydroxylation by CYP8B1, in the NC group. In contrast, significant enrichment of COX reactions was observed in the AS group (Figure 8c). VSMCs exhibited notable accumulation in propanoate metabolism, selenium amino acid metabolism, and tyrosine metabolism (Figure 8d). In both the NC and AS groups, communication between VSMCs and other cell types was activated, with the most abundant receptor-ligand interaction being APP_CD74 in the VSMC|macrophage group. Additionally, the number of APP_CD74 and CCL2_ACKR1 interactions decreased in the VSMC|Endothelial group from the NC to the AS group, whereas interactions involving THBS2_CD36, FN1_integrin_a8b1_complex, FN1_integrin_aVb1_complex, and FN1_integrin_aVb5_complex increased in the VSMC|Macrophage group (Figures 8e,f).

### 3.8 Cluster 9 was identified as the starting point of differentiation

**Figure 9:**
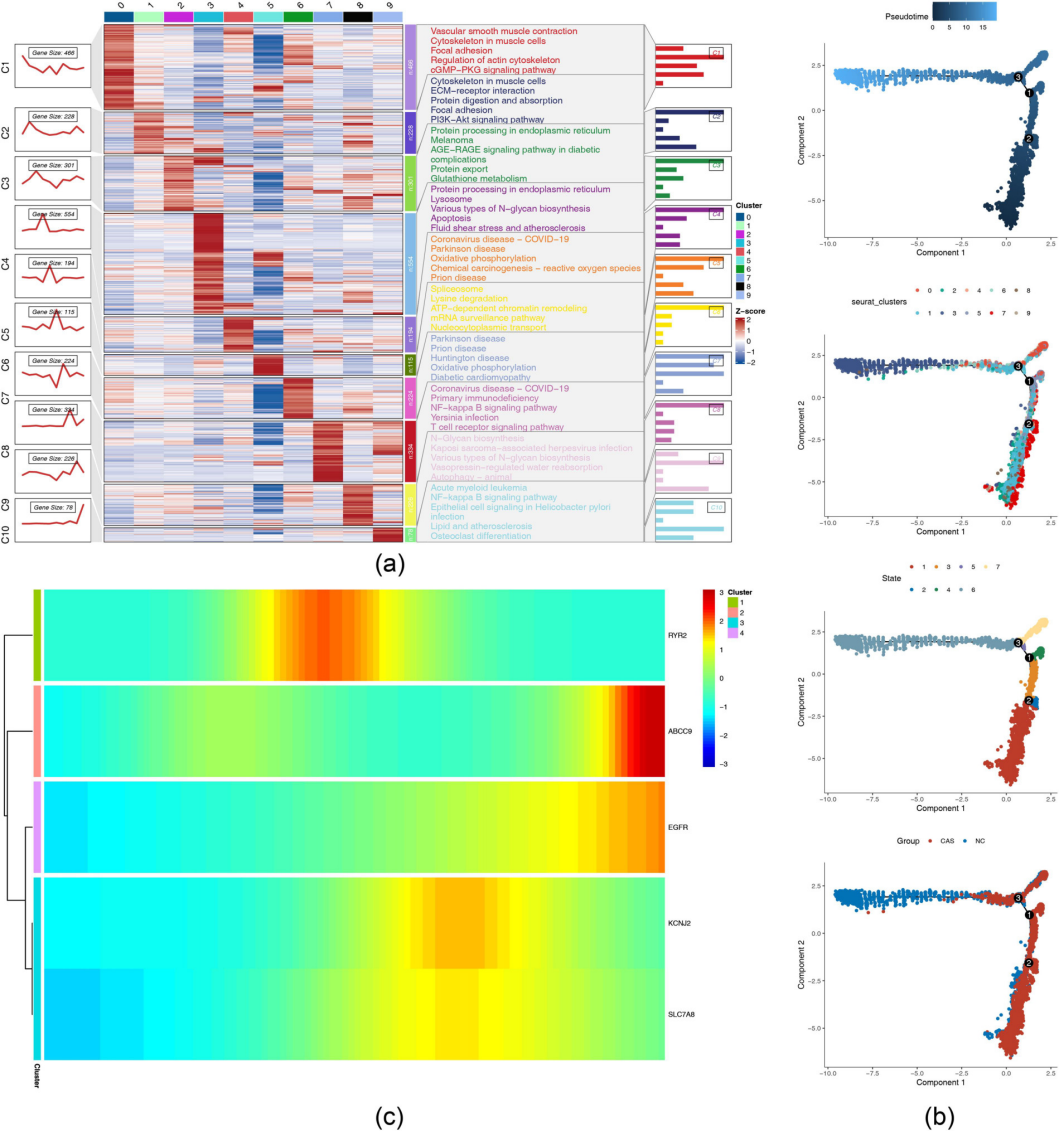
Pseudo-temporal trajectory analysis of atherosclerotic carotid smooth muscle cells (VSMCs) **(a)** enrichment analysis of specific marker genes in different cell subsets of VSMCs. Vertical (row), Represents different gene modules, and each module is a group of genes obtained through cluster analysis. Red indicates that the gene of this module is highly expressed in this cell type. Blue indicates low expression. The left line graph shows the comparison of the expression of specific marker genes for a particular cell subpopulation among all cell subpopulations. The results of KEGG enrichment analysis are shown on the right side; **(b)** The distribution of VSMCs on the differentiation trajectory, from left to right, is as follows, pseudo-time series, cell differentiation subsets, cell differentiation states, cell grouping dynamics. The cell quasi-time series analysis shows the cell conditions divided by the quasi-time axis, with dark parts representing the early stage of the time axis and light parts representing the late stage. The quasi-time series analysis divides cells at different times into seven states; **(c)** Expression heatmap of VSMCs along pseudo-time, where the horizontal and vertical axes represent the pseudo-time series axes, and the depth of color indicates the expression level of the gene, with the vertical axis representing the gene. The data in this figure is sourced from GSE159677.

First, based on the principal component elbow plot (where the first 20 principal components exhibited a significant decrease in variance explained, and the curve flattened after 20 components) and the JackStraw plot (where the first 20 principal components all met the criterion of *p* < 0.05), the first 20 principal components were selected for subsequent analysis (Supplementary Figure S1a,b). Subsequently, after clustering with the FindClusters function (resolution 0.05), VSMCs were divided into 9 distinct cell clusters, and the t-SNE plot clearly illustrated the distribution characteristics of each cluster (Supplementary Figure S1c). KEGG pathway enrichment analysis revealed that Cluster 9 was significantly enriched with early activated pathways in VSMCs, including the MAPK signaling pathway, PI3K-Akt signaling pathway, and NF-kappa B signaling pathway, confirming this cluster as the core VSMC cluster and the starting point of differentiation (Figure 9a). The results showed that during the differentiation and development of VSMCs, a total of three developmental branches and seven differentiation states were identified. Most of the AS cells exhibited a low differentiation state, which may have been due to vascular necrosis caused by lipid deposition (Figure 9b). During VSMC differentiation, the expression levels of ABCC9 and EGFR remained stable from the early to mid-differentiation stages but increased significantly in the late stage. In contrast, the expression levels of RYR2, KCNJ2, and SLC7A8 initially increased during the mid-differentiation stage and subsequently decreased (Figure 9c).

### 3.9 KCNJ2 and SLC7A8 increased and EGFR, RYR2, and ABCC9 decreased in carotid plaques in apoE^–/–^ mice and in hypoxic HASMCs

**Figure 10:**
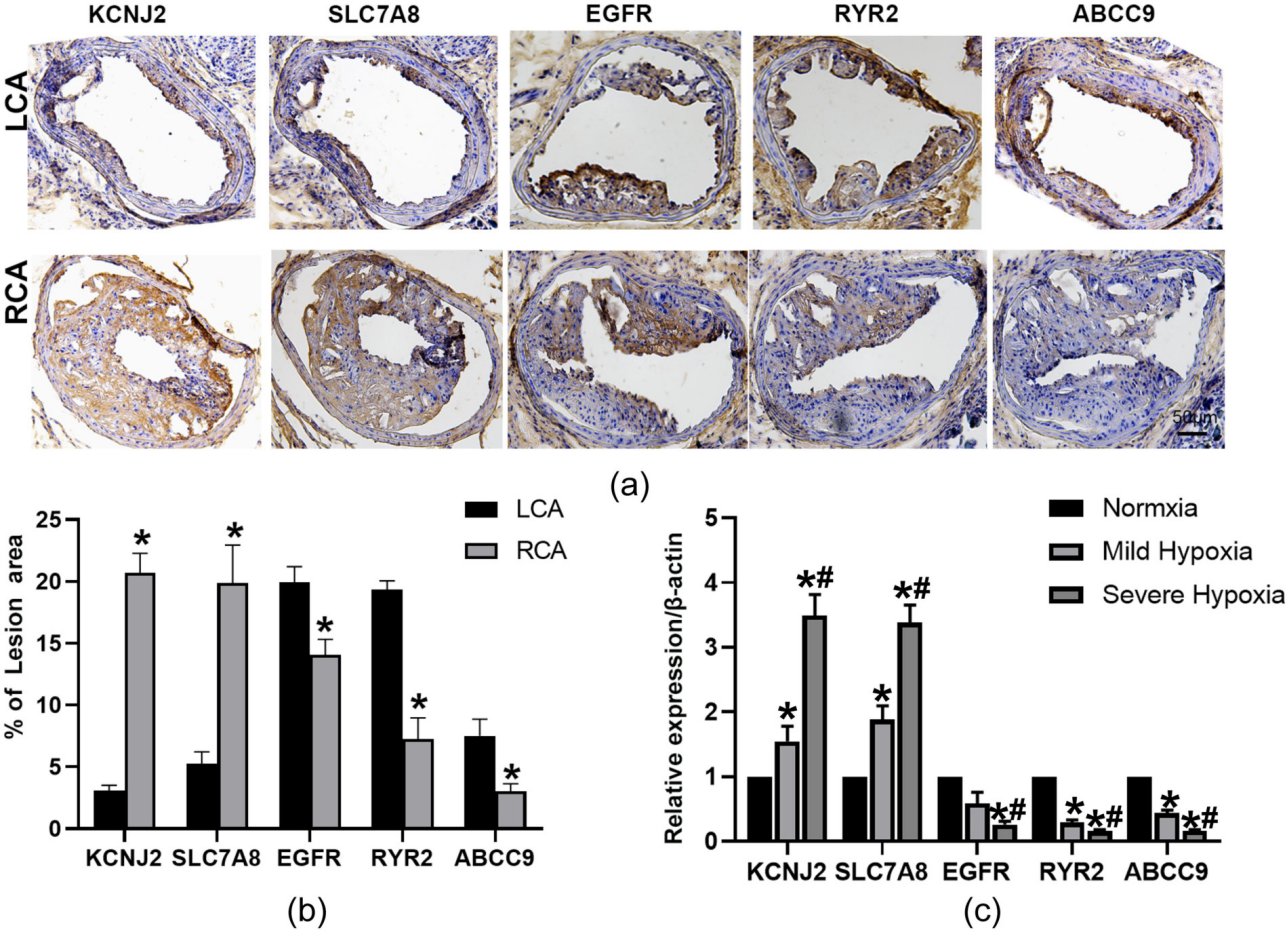
KCNJ2, SLC7A8 increased and EGFR, RYR2, ABCC9 decreased in carotid plaque in apoE-/- mice and in hypoxia HASMCs under stimulation of ox-LDL. **(a)** Representative immunohistochemical stained images of KCNJ2, SLC7A8, EGFR, RYR2, ABCC9 in right and left carotid arteries. Bars represent 50 μm; **(b)** Quantification of KCNJ2, SLC7A8, EGFR, RYR2, ABCC9 in carotid plaque; **(c)** HASMCs were subjected to either normoxic (21% O2), mild hypoxic (10% O2) or severe hypoxic (1%O2)environments 48 h. mRNA levels of KCNJ2, SLC7A8, EGFR, RYR2, and ABCC9 were quantified using RT-qPCR. Data are presented as mean ± SEM, *n* = 3.* *p* < 0.05 compared to LCA or Normoxia; # *p* < 0.05 compared to mild hypoxia.

Relative expression of KCNJ2 and SLC7A8 was higher in the RCA, whereas EGFR, RYR2, and ABCC9 expression was lower in the RCA compared to the LCA (*p* < 0.05) (Figures 10a,b). Compared to HASMCs exposed under normoxic (21% O_2_), mild hypoxic (10% O_2_) and severe hypoxic(1%O_2_)conditions showed gradually increased expression levels of KCNJ2 and SLC7A8 (*p* < 0.05) (Figure 10c). In contrast, expression of EGFR, RYR2, and ABCC9 was gradually descreased in cells with the concentration of oxygen decreases (*p* < 0.05) (Figure 10c).

## 4 Discussion

AS is a multifactorial disease characterized by the accumulation of lipoproteins, immune cells, and other factors (40). Studies suggest that AS develops in regions with elevated hypoxia, which promotes plaque angiogenesis and alters cellular metabolism (41–43). Hypoxia drives angiogenesis within plaques, potentially exacerbating plaque bleeding and progression. It triggers a metabolic shift from aerobic to anaerobic glycolysis for ATP production, mediated by hypoxia-inducible factors (HIFs), thus altering the local metabolic environment (10). Despite its relevance, the role of hypoxia-related metabolic genes in AS remains poorly understood and requires further investigation. This study analyzed HM-RGs using transcriptomic and single-cell data from patients with AS. Hub genes associated with AS, such as RYR2, ABCC9, KCNJ2, EGFR, and SLC7A8, exhibit significant diagnostic potential and are predominantly expressed in VSMCs. The interaction between VSMCs and macrophages appears particularly intricate. VSMCs exhibited a trend of differentiation from right to left, with most AS cells displaying low differentiation states.

From single-cell sequencing and transcriptomic data, 141 DE-MH-RGs were identified as potential hub genes. Enrichment analysis showed these genes to be significantly associated with hypoxia response, lysosomal function, aminoglycan metabolism, glycolysis/gluconeogenesis, cholesterol metabolism, glycosaminoglycan degradation, and pyruvate metabolism. LDL cholesterol (LDL-c) plays a central role in the development of AS, with elevated levels leading to its accumulation in the arterial wall, where oxidation and inflammation occur (44). Additionally, high blood glucose levels can damage ECs, promoting advanced glycation end product (AGE) synthesis, which exacerbates inflammation and vascular injury (45). In AS, lysosomes contribute to lipid degradation and cellular cleaning. Hypoxia impairs lysosomal function, exacerbating AS progression through lysosomal damage and lipid accumulation, which leads to foam cell formation. Hypoxia affects lysosomal lumen acidification and may impair macromolecule degradation. Increased ROS production damages the lysosomal membrane, contributing to cellular damage (46). Under diabetic conditions, ECs undergo metabolic reprogramming, shifting towards glycolysis and suppressing oxidative phosphorylation, which affects proliferation, migration, and angiogenesis. During angiogenesis, glycolysis serves as the primary source of ATP, instead of oxidative phosphorylation. Inhibition of glycolysis can reduce AS (47, 48). Classically activated macrophages primarily rely on glycolysis for proinflammatory activities, while alternatively activated macrophages utilize oxidative phosphorylation for anti-inflammatory functions (48). Pyruvate dehydrogenase kinases PDK1 and PDK4 promote vascular inflammation and plaque vulnerability (49). Identifying new metabolic dysfunction targets within plaque cells holds considerable promise for improving vulnerable plaques and reducing cardiovascular events.

Further screening identified five hub genes (KCNJ2, SLC7A8, EGFR, ABCC9, and RYR2) with high diagnostic potential for AS. Expression levels of RYR2, ABCC9, and EGFR were downregulated in carotid arteriosclerosis tissues, while KCNJ2 and SLC7A8 showed upregulation in the same tissues.

KCNJ2 (Kir2.1) encodes a potassium channel protein present in the heart and other tissues. Reduced expression of KCNJ2 inhibits neointimal formation after carotid artery injury in rats (50). Silencing KCNJ2 decreases lipid content and total cholesterol levels in THP-1 cells (51). Moreover, endothelial KCNJ2 significantly influences flow-induced vasodilation and lesion formation in hypercholesterolemic ApoE^–/–^ mice (52). The role of KCNJ2 in AS is likely multifaceted across various cell types, but its specific function in AS pathogenesis remains unclear.

SLC7A8 (LAT2) facilitates amino acid transport and its deficiency prevents diet-induced obesity, reduces glucose intolerance, limits lipid accumulation in multiple organs, and diminishes macrophage infiltration (53). SLC7A8 regulates protein translation and cell proliferation through the mTORC1 pathway, enhancing glycolysis in several cancers (54, 55). These findings suggest a potential positive correlation between SLC7A8 and cellular proliferation, metabolism, and glycolysis, though its role in AS remains uncertain.

Epidermal growth factor receptor (EGFR) plays a critical role in AS development by promoting cell proliferation, migration, infiltration, apoptosis inhibition, and inflammation regulation (56, 57). Our analysis revealed decreased EGFR expression in carotid plaques, possibly related to EC apoptosis in advanced arterial plaques. Further research is needed to elucidate the underlying mechanisms.

Cassette subfamily C member 9 (ABCC9) is part of ATP-sensitive potassium ion channels (58). Mutations in ABCC9 impair cardiac stress adaptation, leading to myocardial damage and are associated with diabetes and obesity (59, 60). ABCC9^–/–^ mice exhibit elevated resting blood pressure, ST segment elevation, and coronary vasospasm (61). These findings suggest that ABCC9 protects cardiomyocytes, influences EC diastolic function, and supports normal glucose and lipid metabolism, potentially exerting anti-atherosclerotic effects.

The type 2 ryanodine receptor (RYR2), located in the endoplasmic reticulum, mediates intracellular calcium ion release through calcium release channels (62). Ox-LDL and hypoxia impact RYR2 activity and calcium homeostasis, leading to oxidative stress and inflammation. However, RYR2 binds to calmodulin to mitigate AS (63, 64). In VSMCs, RYR2 is critical for maintaining normal blood vessel function (63). It reduces the risk of plaque rupture by inducing a systolic phenotype in VSMCs (65). RYR2 has also been identified as a correlate of immune cell infiltration in AS (66), although its specific mechanisms remain under investigation.

Among the key cellular components, the five hub genes exhibited elevated expression in VSMCs and were significantly linked to histidine metabolism, as well as the metabolism of alanine, aspartic acid, and glutamic acid.

The inflammatory response within the aortic intima involves multiple cell types, including VSMC subtypes, ECs, macrophages, and T and B lymphocytes (67). VSMCs, the dominant cell type in the medial layer of the arterial wall, are essential for maintaining vascular tone and producing the extracellular matrix. Phenotypic changes and altered antioxidant status of VSMCs are critical in the progression of AS (68). The transition from a contractile to a synthetic phenotype plays a key role in neointimal hyperplasia and the development of AS. VSMCs can become foam cells after exposure to aggregated or ox-LDL in the intima and media of hyperlipidemic mice and humans. Through migration, proliferation, senescence, and secretion of matrix proteins and proinflammatory mediators, VSMCs contribute to both atherogenesis and plaque stability (69, 70). VSMCs also play a significant role in oxidative stress and inflammatory pathways, which substantially influence the development of conditions such as AS and pulmonary hypertension (71, 72). Macrophages activate VSMCs by releasing proinflammatory cytokines and chemokines, while foam cells derived from macrophages secrete growth factors and cytokines that promote VSMC proliferation, migration, and plaque progression (73). Additionally, macrophages regulate lipid metabolism in VSMCs, influencing lipid uptake and foam cell formation (74). Our findings indicate that most AS cells exhibit limited differentiation, and the number of VSMCs declines during AS progression, possibly due to lipid-induced vessel necrosis. These results suggest that hub genes may regulate VSMC metabolism, impacting AS pathogenesis and offering potential therapeutic targets for AS treatment.

Pseudotime analysis tracks temporal changes in individual cells, revealing shifts in gene expression and providing insights into biological processes such as cell growth, specialization, and activity. This method enhances the understanding of individual cell data and facilitates the identification of novel biological insights and biomarkers (75). In the present study, the initiation point of cell differentiation and the trajectory of cell state transitions were visualized. Pseudotime analysis was performed on the NC and AS groups, generating trajectory plots that depict transcriptional states associated with cell development. The expression of five hub genes, located at key loci related to cell differentiation, was also elucidated. The results indicated that VSMCs in the disease environment exhibited a continuous differentiation spectrum characterized by multiple nodes and states, with the majority of AS cells tending to cluster in a less-differentiated state. This phenomenon may suggest that pathological microenvironmental factors within the plaque, such as lipid deposition, might drive VSMCs to enter a state of functional dormancy or de-differentiation, thereby potentially affecting the plaque's reparative capacity and stability. Notably, the five hub genes displayed distinct dynamic expression patterns along this differentiation trajectory. The upregulation of ABCC9 and EGFR in the later stages of differentiation suggests they might play a role in the late phase of the VSMC response to persistent pathological stimuli, for instance, by participating in cellular adaptive survival or the regulation of terminal differentiation signaling. Meanwhile, the expression trend of RYR2, KCNJ2, and SLC7A8—characterized by an initial upregulation followed by downregulation during the mid-stage of differentiation—suggests that these genes are likely involved in the phenotypic switching of VSMCs.

While this study explored the potential roles of hypoxia and metabolism in atherosclerosis (AS) through bioinformatics and experimental validation, recent research suggests that gastroesophageal reflux disease (GERD) may be an overlooked, system-wide hypoxia-related risk factor for AS. A study by R. Salvador et al. confirmed that GERD events, particularly proximal reflux, are closely associated with significant oxygen desaturation, providing direct evidence that GERD induces transient hypoxia (76). This GERD-triggered intermittent hypoxia pattern may synergize with chronic intra-plaque hypoxia to induce oxidative stress and systemic inflammation, thereby driving AS progression. This viewpoint is supported by clinical research from Ziyang Wu et al., whose predictive model confirmed that GERD patients are a high-risk population for coronary atherosclerosis, establishing a direct epidemiological link between GERD and AS (77). Therefore, we speculate that GERD may induce intermittent hypoxia, which in turn leads to metabolic/inflammatory disorders, thereby promoting the development and progression of AS. The key genes identified in our study (RYR2, ABCC9, KCNJ2, EGFR, SLC7A8), which function as regulators of hypoxia and metabolism, are likely to play a role in this process. Consequently, while future research should continue to deeply investigate the role of these key genes in AS, it is also crucial to explore whether GERD-related hypoxia influences the atherosclerotic process by regulating these hub genes. Such studies would not only enrich our understanding of the systemic triggers of AS but may also open new avenues for cross-system joint intervention strategies.

## 5 Conclusions

In this study, AS was found to be linked to metabolic hypoxia, and the hub genes RYR2, ABCC9, KCNJ2, EGFR, and SLC7A8 were identified as strong diagnostic markers. Key metabolic processes involved in hypoxia, such as aminoglycan metabolism, lysosomal composition, metal ion transmembrane transporter activity, and glycolysis, were implicated. The highest expression levels of RYR2, ABCC9, KCNJ2, EGFR, and SLC7A8 were observed in VSMCs within AS. The communication between VSMCs and macrophages was notably interlinked via APP_CD74. In the AS group, VSMCs exhibited a trend of differentiation from right to left, while the majority of cells demonstrated a low degree of differentiation. This study is the first to examine changes in cell, gene, and pathway expression related to hypoxia and metabolism in AS, specifically in VSMCs, through single-cell RNA sequencing. The findings highlight the critical role of metabolic shifts, such as glycolysis, in AS progression and point to potential new hub genes. These results provide key targets for further research and offer significant implications for personalized treatment strategies for patients with AS. However, this study has several limitations. First, concerning the translational relevance, the hub genes were identified using human carotid artery samples but were validated *in vivo* using the ApoE^−/−^ mouse model. While the ApoE^−/−^ model is the classical tool for atherosclerosis research, we explicitly acknowledge the inherent species-specific differences in metabolic, inflammatory, and plaque stability profiles compared to human pathology. We highlight that the consistent expression patterns across both species suggest an evolutionarily conserved mechanism; nevertheless, future studies are warranted to further bridge this gap using human atherosclerotic plaques or organoid models. Second, The small sample size of the scRNA-seq dataset may introduce potential bias, thus affecting the generalizability of our findings. Third, the complex molecular mechanisms through which these hub genes influence the pathogenesis of AS remain to be fully clarified. Further functional studies are necessary to define their precise roles in cell-type-specific contexts.

## Data Availability

The original contributions presented in the study are included in the article/Supplementary Material, further inquiries can be directed to the corresponding author.
