# Supplementary figures and images for: Identifying and validating hypoxia- and metabolism-related hub genes and cell communication in atherosclerosis
Source: Front Cardiovasc Med. 2025 Dec 12;12:1680482. doi: 10.3389/fcvm.2025.1680482 (PMC12740931; doi:10.3389/fcvm.2025.1680482)

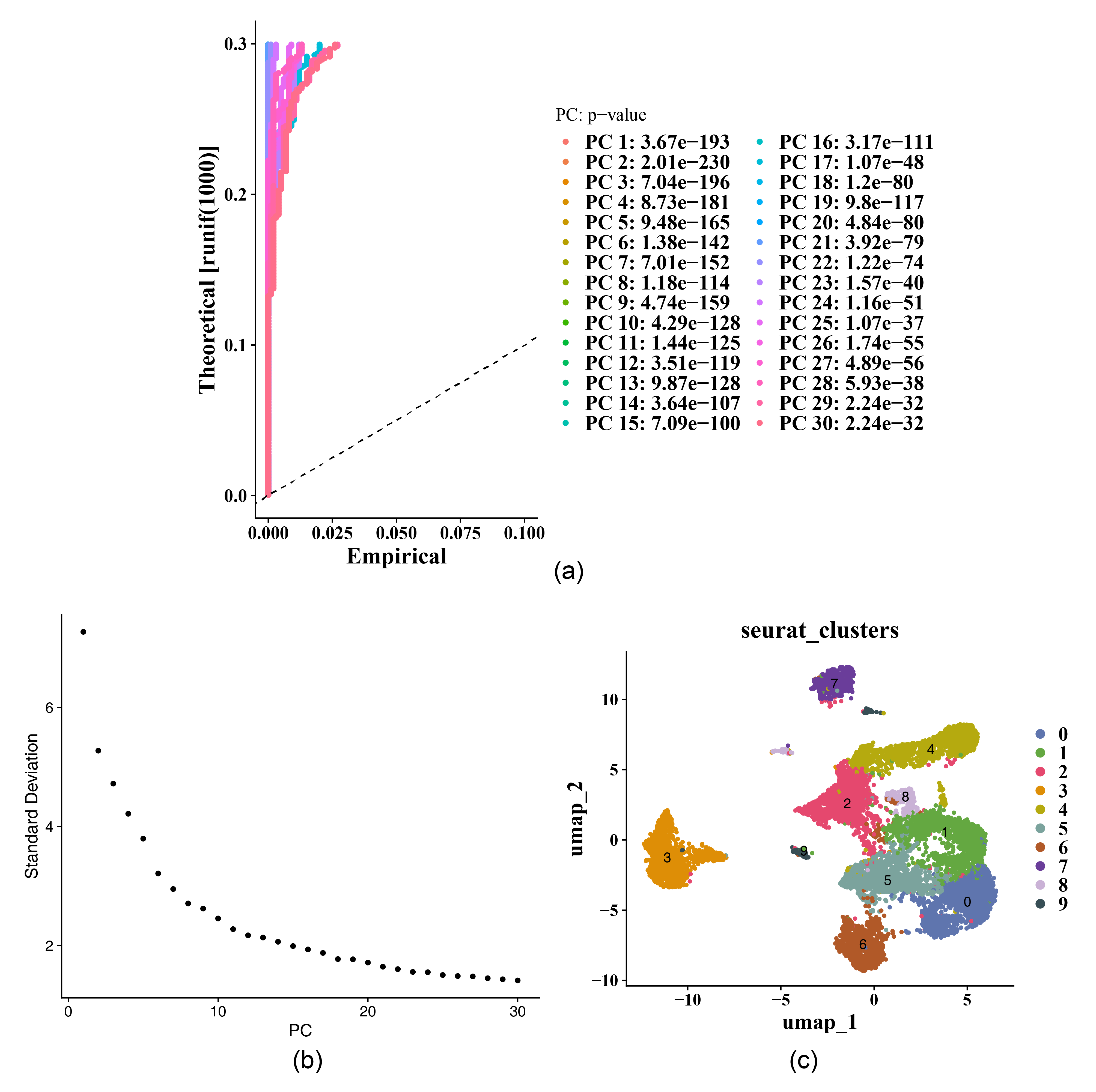

Supplement: Supplementary file 1 [file Image1.tif]
